# The pathway of social support in enhancing adolescents’ physical fitness: The mediating roles of physical activity and self-efficacy

**DOI:** 10.1371/journal.pone.0308864

**Published:** 2024-09-26

**Authors:** Zhiyong Xu, Jinwen Xu, Tianshu Liu, Zhiyang Gu, Donglin Hu

**Affiliations:** 1 Jiangsu Vocational Institute of Commerce, School of Physical Education, Nanjing, China; 2 College of Physical Education, Yangzhou University, Yangzhou, China; 3 School of Sports Science and Engineering, East China University of Science and Technology, Shanghai, China; 4 Experimental Centre for Exercise and Health Promotion, Nanjing Agricultural University, Nanjing, China; Huashan Hospital Fudan University, CHINA

## Abstract

**Purpose:**

Utilising Welk’s Youth Physical Activity Promotion (YPAP) model as a foundational framework, this study investigates the intricate interplay of social support, physical activity (PA), and self-efficacy in relation to physical fitness within the context of Chinese culture. The primary objective is to identify the nuanced dynamics among social support, self-efficacy, PA, and physical fitness to enhance adolescent well-being and fitness.

**Methodology:**

The study employed a convenience sampling method, engaging 123 adolescents aged 18–21, of which 67 were females (54.47%), and 56 were males (45.53%). Data were collected through structured questionnaires focusing on the identified variables.

**Results:**

Our study revealed significant positive associations among social support, self-efficacy, PA, and physical fitness, with correlation coefficients ranging from 0.282 to 0.419. Notably, a discernible gender disparity emerged, with females exhibiting higher levels of physical fitness. Among the key determinants of adolescent physical fitness, self-efficacy emerged as the most influential, followed by PA and gender. Utilising structural equation modelling and regression techniques, we discerned that social support indirectly influences physical fitness, primarily mediated by self-efficacy and the level of physical activity.

**Discussion:**

This study provides insight into how social support impacts adolescent physical fitness. We found that social support strongly predicts both PA and self-efficacy, and self-efficacy significantly boosts PA, ultimately leading to improved physical fitness. Both self-efficacy and PA serve as mediators in the relationship between social support and fitness. Therefore, interventions should prioritise reinforcing social support, promoting PA, and nurturing self-efficacy to optimise adolescent physical fitness outcomes.

## 1. Introduction

In today’s rapidly changing societal panorama, profound transformations in human structures and lifestyles have provided greater means to basic human needs. However, this progress has inadvertently led to a notable decrease in physical activity, ushering in an era of sedentary living [1]. Extensive scholarly investigations lauds the merits of regular physical engagement, emphasising its pivotal role in weight management, cardiovascular resilience, mental well-being, academic achievements, and the development of adolescents’ individual accountability and collaborative spirit [2–4].

Recent findings highlight a concerning trend: an annual 7% decline in physical activity (PA) among the global adolescent population. If sustained, this trend could result in a staggering 60% to 70% plunge as these individuals transition into adulthood [5]. Authoritative health institutions in China and several industrially advanced Western countries recommend dedicating at least three sessions per week, each lasting an hour, to moderate-to-vigorous physical activity (MVPA), viewing it as a fundamental requirement for maintaining physical fitness [1,6]. Alarmingly, only 30% of Chinese students and young adults faithfully adhere to these endorsed prescriptions [7].

Such a shortage of youthful physical endeavour poses a risk to physical vigour and may cast a shadow over their academic endeavours and overall quality of life. Available data documents a two-decade-long decline in the physical robustness of Chinese youth, underscoring a pressing call for remedial actions [8]. With a staggering 44.3 million university students in China [7], enhancing the physical fitness of this group is not merely an issue of individual well-being; it is a matter of national public health as well. Hence, understanding and addressing the factors influencing adolescents’ physical fitness is of paramount importance.

Numerous scholars have investigated the factors influencing adolescent PA to modify these determinants and enhance PA levels, subsequently improving adolescent physical fitness. While contemporary research predominantly underscores the significance of social support in invigorating adolescent physical activity [9–11], it is imperative to recognise that the tapestry of adolescent PA behaviours is intricate. Relying solely on social support as the cornerstone falls short’ self-efficacy holds substantial sway in this realm [4,12]. Invoking Bandura’s social cognitive theory, it becomes evident that self-efficacy and social support play instrumental roles in shaping individual behaviours [13]. When complemented by substantial social support, adolescents fortified with high levels of self-efficacy typically exhibit a firm belief in their behavioural capabilities and are more inclined towards health-oriented actions.

Moreover, the influence of social support is not merely supplemental; it actively nurtures and elevates self-efficacy among adolescents [10]. By facilitating triumphant experiences, social support acts as a fulcrum, bolstering an adolescent’s self-efficacy [14]. In essence, a nurturing social backdrop inspires adolescents to engage in sports and amplifies their inherent belief in their sports-related abilities.

Welk [15] introduced the Youth Physical Activity Promotion (YPAP) model, which integrates socio-ecological and social cognitive theories, providing insight into how health can be improved through specific behaviours. This model posits that an adolescent’s physical fitness correlates with the following factors:

Enabling Factors: These encompass an individual’s inclination towards PA, activity level, and motor skills.

Predisposing Factors: These involve psychological elements such as attitudes, values, beliefs, and insights. An individual’s preference, self-efficacy, enjoyment, and comprehension of PA fall under this category.

Reinforcing Factors: These pertain to external encouragement an individual receives, whether directly from family or peers or indirectly through societal evaluations.

**Fig 1:**
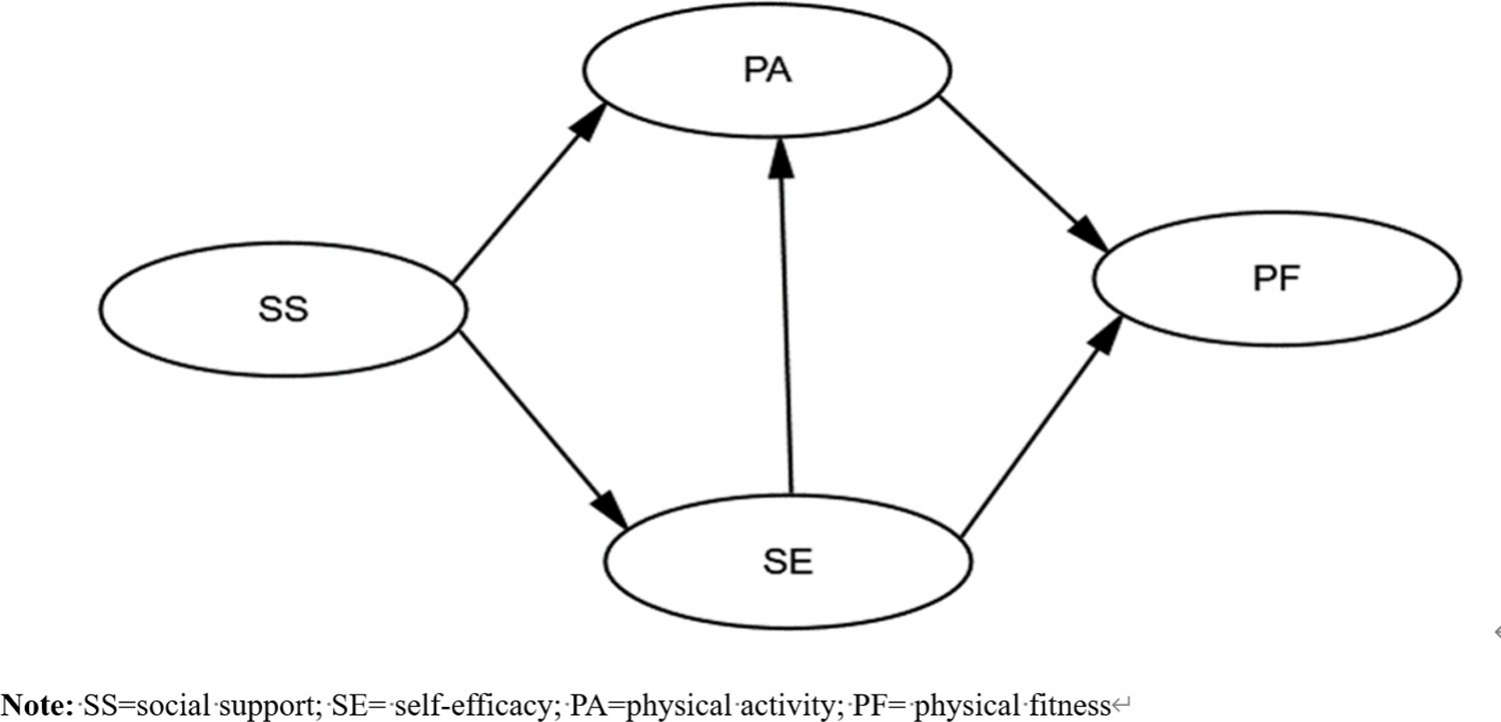
Path diagram illustrating the influence of social support on adolescent physical fitness.

The YPAP model offers a conceptual framework that clarifies how social support may influence physical fitness, with self-efficacy acting as a key mediator in determining individual physical regimens [15,16]. Notably, the applicability and nuances of this linkage in the Chinese context require further investigation. Understanding the dynamic interplay between these variables holds significant implications for health policy formulation in China. Our study aims to fill this gap by exploring the enhancement of adolescent physical fitness through social support. We particularly emphasise the interconnected relationships among social support, self-efficacy, PA, and levels of physical fitness. Drawing upon an integration of existing research and theoretical frameworks, we have formulated a research model, as depicted in Fig 1. In this model, social support is positioned as the independent variable, while PA and self-efficacy serve as mediators, leading to the dependent outcome: physical fitness. This initiative represents one of the first attempts to assess the indirect effects of social support on fitness within the context of the YPAP model, highlighting the crucial roles of PA and self-efficacy.

This study seeks to delineate the pathways through which social support enhances the physical fitness levels among adolescents. Based on our research objectives, we have formulated the following hypotheses:

H1: Social support positively correlates with PA.
H2: Social support positively correlates with self-efficacy.
H3: PA positively correlates with the level of physical fitness.
H4: Self-efficacy positively impacts PA.
H5: Self-efficacy positively correlates with the level of physical fitness.
H6: Social support indirectly impacts the physical fitness level of adolescents via PA.
H7: The influence of social support on adolescent physical fitness is mediated through self-efficacy.
H8: Social support indirectly influences adolescents’ physical fitness through both self-efficacy and PA.

## 2. Methodology

### 2.1 Participants

Participants for this study were selected from a prominent university situated in Nanjing, Jiangsu Province, China. To ensure a diverse representation across disciplines, the university enlisted students from both the liberal arts and sciences, including fields such as literature, history, philosophy, engineering, physics, and computer science. The sample consisted of 123 adolescents aged 18 to 21 years. A convenience sampling method was utilised for participant recruitment. Although this method has limitations and may introduce biases by relying on the availability and willingness of participants rather than random selection, it was necessary for quick and easy recruitment. Of the 170 questionnaires distributed, a total of 135 were returned with valid responses, resulting in a response rate of 79.41%. After excluding 12 incomplete questionnaires, the final dataset comprised 123 complete responses. In terms of gender distribution, the sample was fairly balanced: 67 females (54.47%) and 56 males (45.53%). This study was conducted in compliance with the principles outlined in the Declaration of Helsinki. Ethical approval was granted by the Ethics Committee of Tianjin University of Sport, China (2023–011, 15/03/2023). In this study, written informed consent was obtained from all participants. As the study did not involve any minors, parental or guardian consent was not required. The recruitment period for this study began on 20/03/2023 and concluded on 31/03/2023.

### 2.2 Physical activity measurement instrument

The Physical Activity Questionnaire for Adolescents (PAQ-A) was employed to assess the physical activity levels of adolescents. The Chinese version of PAQ-A has proven to have strong internal consistency and satisfactory validity, establishing it as a reliable tool for large-scale studies [17]. Widely adopted for evaluating adolescent physical activity in China, the PAQ-A was applied to a diverse Chinese adolescent sample using a 5-point Likert scale. For example, how many times did you actively participate in physical exercise or other extracurricular sports activities last weekend? [Single choice] ○ None ○ 1 time ○ 2–3 times ○ 4–5 times ○ More than 5 times. The 5 options correspond to 1 to 5 points on the Likert scale. The total score is an average of the first eight questions, making it suitable for extensive cross-sectional surveys. The Cronbach alpha coefficient for PA in this sample was 0.835.

### 2.3 Social support measurement instrument

This study utilised the Social Support Rating Scale (SSRS) developed by Prof. Xiao Shuiyuan, a Chinese psychologist [18]. Appreciated for its simplicity, reliability, and versatility, the SSRS is designed to measure various aspects of daily life social support. It encompasses three dimensions:

Subjective support (4 items): Perceived support from others.

Objective support (3 items): Tangible support available to the individual.

Support Needs (3 items): The degree to which an individual relies on others for support.

For example, how many close friends do you have that you can count on for support and help? (Single choice) (1) None (2) 1–2 friends (3) 3–5 friends (4) 6 or more friends. The options 1 to 4 in the scale correspond to 1 to 4 points on the Likert scale. A higher score indicates greater social support. Scores are categorized as below 20 (low), 20–30 (average), and 30–40 (satisfactory). The SSRS effectively evaluates the quality of daily social support, offering valuable insights for psychological interventions. The Cronbach’s alpha coefficient for SSRS in this study was an impressive 0.889.

### 2.4 Self-efficacy scale

The adapted General Self-Efficacy Scale (GSES) by Prof. Ralf Schwarzer was used in this study. Initially a 20-item scale, it was refined to 10 items. In collaboration with Chinese scholar Zhang Jianxin, a version tailored for Chinese participants was developed, retaining high reliability and validity [19,20]. Using a four-point Likert scale, the average score, ranging from 1 to 4, indicates a participant’s level of self-efficacy. For example, participants responded to the statement "I can cope with the difficulties I may encounter in my life" using a four-point Likert scale: 1 for strongly disagree, 2 for disagree, 3 for agree, and 4 for strongly agree. Scores below 2.5 suggest low self-efficacy. The GSES demonstrated a Cronbach’s alpha coefficient of 0.750 in this research.

### 2.5 Physical fitness test

In November 2022, our university conducted an in-depth study to assess the physical fitness of students, in strict accordance with the National Physical Fitness Standard for Students (2014 Revision) set forth by China’s Ministry of Education. This endeavor embodies the university’s commitment to understanding and enhancing students’ physical fitness, aligning with the nationwide initiative to assess youth physical fitness of the youth in accordance with educational benchmarks across the country.

The assessment utilised a diverse range of metrics designed for a comprehensive appraisal of fitness. These included sensitivity, measuring students’ coordination and response time; flexibility, gauging joint and muscular mobility; and cardiorespiratory endurance, assessing the efficiency of the heart and lungs during sustained exertion.

To ensure accuracy, specially trained physical education instructors administered the tests, guaranteeing that the results genuinely reflected the students’ capabilities. The cumulative score, calculated from a weighted sum of these metrics, peaks at 100 points. A threshold of 60 marks the pass rate, emphasising the high standard of fitness expected within the academic community.

### 2.6 Data processing

The data were processed using SPSS 26.0 (IBM Corporation, Armonk, New York) and AMOS 24.0 (IBM Corporation, Armonk, New York), with the validity of the scales confirmed prior to their application. Descriptive statistics entailed the calculation of mean and standard deviations. Pearson’s correlation was employed to uncover relationships among variables, paving the way for regression analyses. Stepwise regression incorporated gender, social support, self-efficacy, and PA as independent variables against physical fitness as the dependent variable, thereby ascertaining the predictors of physical fitness. Following the SPSS analysis, AMOS 24.0 was employed for structural equation modelling (SEM), examining the interrelations between social support, self-efficacy, PA, and physical fitness.

## 3 Results and analysis

### 3.1 Descriptive statistics

Descriptive statistics were used to analyse scores pertaining to social support, self-efficacy, PA, and physical fitness. These scores were represented by means (M) and standard deviations (SD). The findings revealed an average social support score of 2.48, indicating a general level of social support. The mean self-efficacy score was 2.51, hovering near the critical threshold of the GSES scale. Notably, scores below 2.5 were indicative of low self-efficacy. The average score for PA stood at 3.02, surpassing the midpoint of 2.5, and the mean physical fitness score was 68.56, exceeding the passing benchmark of 60. The linear association aptly quantifies the interplay between variables. As delineated in Table 1, a significant positive correlation was observed among social support, self-efficacy, PA, and physical fitness, with correlation coefficients ranging from 0.282 to 0.419. It is evident that as social support intensifies, there’s a concomitant increase in self-efficacy and PA, leading to improved physical fitness outcomes.

**Table 1:** Descriptive statistics and correlation analysis results of each variable.

Variable (n = 123)	M	SD	SS	SE	PA	PF
**SS**	2.48	0.08	1	0.282**	0.408**	0.287**
**SE**	2.51	0.06	0.282**	1	0.306**	0.419**
**PA**	3.02	0.07	0.408**	0.306**	1	0.362**
**PF**	68.56	0.55	0.287**	0.419**	0.362**	1

Note: SS = social support; SE = self-efficacy; PA = physical activity; PF = physical fitness.

** Correlation is significant at the 0.01 level (2-tailed) * Correlation is significant at the 0.05 level (2-tailed).

### 3.2 Regression analysis: The interplay of social support, self-efficacy, PA, and physical fitness in adolescents

To account for gender effects, the categorical gender variable was transformed into a dummy variable, using females as the reference category. The objective of our regression model was to elucidate the influence of several variables, including social support, self-efficacy, gender, and PA level, on adolescent physical fitness. Notably, self-efficacy emerged as the most influential predictor of adolescent physical fitness, followed by PA, and then gender. In contrast, social support did not yield a discernible impact on physical fitness and was consequently excluded from the model. Given the number of predictors and the sample size, these independent variables accounted for 24.5% of the variance in the outcome. The unstandardised regression coefficients for self-efficacy, PA, and gender stood at 3.021, 2.095, and 2.083, respectively. It is important to highlight that the influence of gender on physical fitness underscored a significantly higher level of fitness among girls compared to boys (Table 2).

**Table 2:** Regression analysis of social support, self-efficacy, PA, and physical fitness.

	Dependent variable	
	PF	
	(1)	(2)
SS	0.608 (0.625)	
SE	2.892** (0.838)	3.021** (0.828)
PA	1.845** (0.729)	2.095** (0.683)
Sex	1.967* (0.995)	2.083* (0.988)
Constant	53.155 (2.495)	53.526** (2.465)
Observations	123	123
R^2^	0.269	0.263
Adjusted R^2^	0.245	0.245
Residual Std.Error	5.342 (df = 118)	5.341 (df = 119)
F Statistic	10.875**	14.190**

*Note* *p<0.05

**p<0.01.

Note: SS = social support; SE = self-efficacy; PA = physical activity; PF = physical fitness.

### 3.3 Examining mechanisms: The mediating role of PA and self-efficacy in the relationship between social support and physical fitness

**Fig 2:**
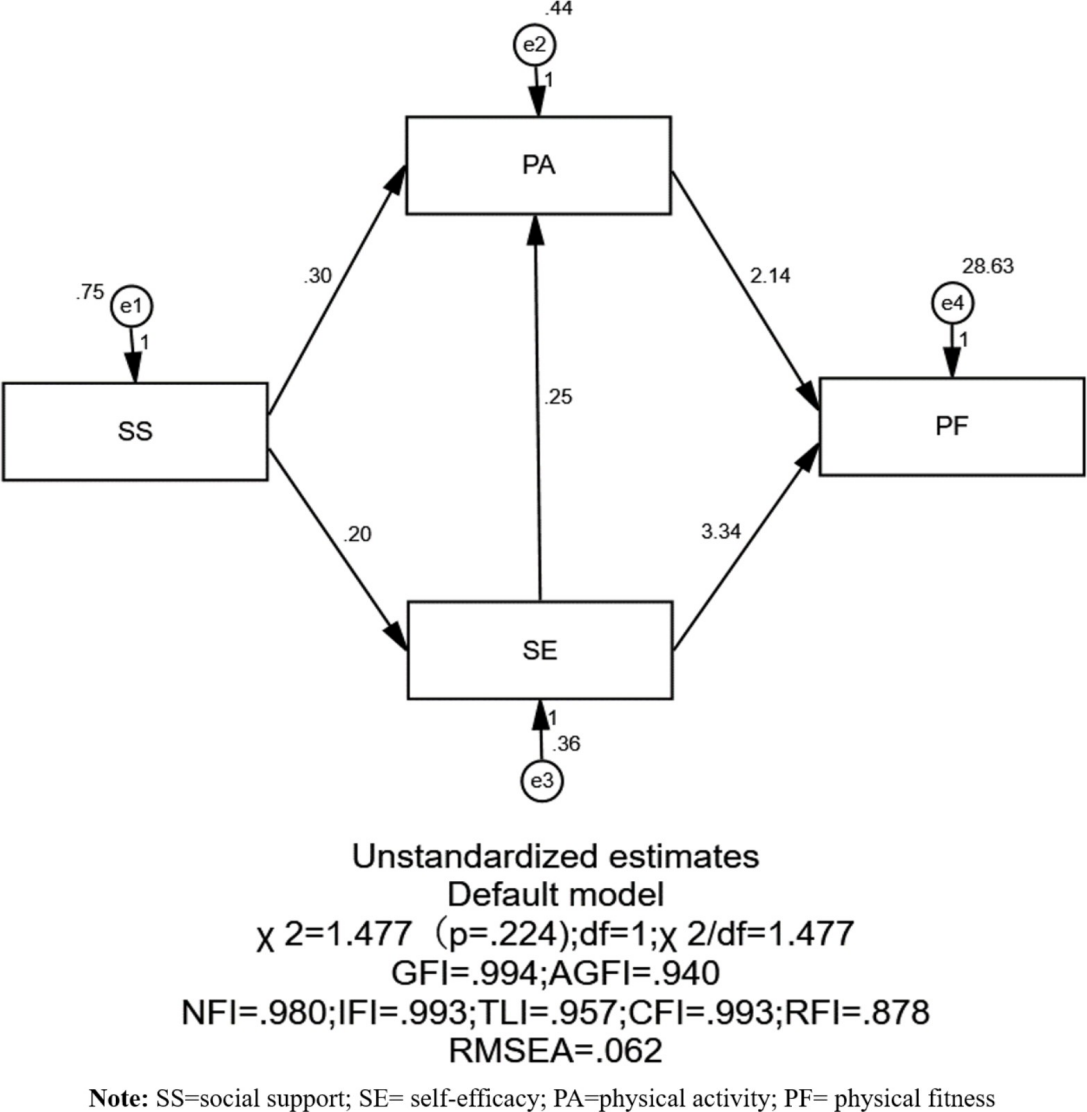
Diagram illustrating the SEM’s pathway linking social support and adolescent physical fitness.

Using SEM in conjunction with correlation and regression analyses, we aimed to elucidate the underlying mechanism linking social support to physical fitness. Model estimations and hypothesis tests were conducted using the Maximum Likelihood method. Fig 2 displays the model’s fit indices: *X*^*2*^*/*df = 1.477, RMSEA = 0.062, GFI = 0.994, AGFI = 0.940, NFI = 0.980, IFI = 0.993, with a standardised RMR of 0.027. Additionally, TLI = 0.995, CFI = 0.993, and RFI = 0.879. Collectively, these metrics suggest a commendable model fit.

A multiple mediation model was constructed using Hayes’ PROCESS (version 3.4) with a 95% confidence interval. As asserted by Hair, Anderson [21], bootstrap samples ideally should exceed the the number of valid observations in the original dataset, and they recommended 5,000 iterations. Thus, this study adopted 5,000 bootstrap samples. The model assessed the potential mediating roles of PA and self-efficacy between social support and physical fitness. Results confirmed all direct effect hypotheses (H1-H5). Specifically, both social support (b = 0.299; 95% CI = 0.145, 0.450) and self-efficacy (b = 0.247; 95% CI = 0.045, 0.456) exert a direct influence on PA. Additionally, social support has a direct effect on self-efficacy (b = 0.203; 95% CI = 0.083, 0.331). Furthermore, PA (b = 2.136; 95% CI = 0.540, 3.524) and self-efficacy (b = 3.344; 95% CI = 1.675, 4.972) each directly impact physical fitness. In evaluating the mediation effects, both PA (b = 0.640; 95% CI = 0.146, 1.380) and self-efficacy (b = 0.679; 95% CI = 0.227,1.445) emerged as significant mediators between social support and adolescent physical fitness, as detaliled in Table 3. Consequently, hypotheses H6 and H7 were validated. Moreover, the mediation roles of self-efficacy and PA remained significant for the pathway represented by H8 (b = 0.107; 95% CI = 0.025, 0.317), confirming the validity of hypothesis H8.

**Table 3:** Analysis of path coefficients among variables.

No.	Variable	Estimate/β	Lower	Upper	S.E	C.R/*t*	*p-*Value
H1	SS→PA	.299	.145	.450	.072	4.151	**
H2	SS→SE	.203	.083	.331	.063	3.247	**
H3	PA→PF	2.136	.540	3.524	.687	3.111	**
H4	SE→PA	.247	.045	.456	.100	2.466	*
H5	SE→PF	3.344	1.675	4.972	.818	4.086	**
H6	SS→PA→PF	.640	.146	1.380	-	-	**
H7	SS→SE→PF	.679	.227	1.445	-	-	**
H8	SS→SE→PA→PF	.107	.025	.317	-	-	**

**Note:** **: *p* <0.01

*: *p* <0.05, SS = social support, SE = self-efficacy, PA = physical activity, PF = physical fitness, Indirect effects and 95% confidence interval with 5000 bootstraps, predicting physical fitness (n = 123) Lower = lower limit confidence interval, Upper = upper limit confidence interval.

## 4 Discussion

This study explores the mechanisms by which social support enhances the physical fitness of adolescents. Our focus was on understanding the interplay between social support, self-efficacy, PA, and physical fitness. The findings provide empirical support for Welk’s model of adolescent PA promotion, particularly within a Chinese context. Drawing on the YPAP model, this study is a pioneering endeavour to discern the mediated impact of social support on physical fitness levels through PA and self-efficacy.

### 4.1 The influence of social support on PA and self-efficacy

The role of social support in influencing PA and self-efficacy among adolescents has drawn increasing scholarly attention. Our study posited a positive relationship between social support and PA, a hypothesis that was empirically validated. Specifically, the data revealed that adolescents with substantial social support exhibited a greater inclination towards PA. This aligns with a growing body of research advocating that enhanced social support simulates increased engagement in physical exercise, thereby fostering health-positive behavioural shifts. It Noteworthy is that sustained social support serves as a bulwark for perseverance in physical regimens, empowering individuals to overcome obstacles and maintain consistent engagement. Such revelations accentuate the pivotal importance of social support in driving PA, positioning it as a potential cornerstone in efforts to enhance public health. This perspective is echoed in international inquiries, with research by Ren, Hu [10] and Pham, Wawrzyniak [3] pinpointing social support as a significant determinant of adolescent PA. A deeper exploration by Kastrati, Gashi [9] draws attention to the essential influence of parental figures, shedding light on diverse modalities through which they can inspire PA in their children. From a programmatic standpoint, it becomes evident that initiatives seeking to enhance student PA should underscore the intrinsic value of social support.

Transitioning to the examination of the relationship between social support and self-efficacy, our research posited a positive correlation, a postulation substantiated by our empirical observations. This synergy aligns seamlessly with the prevailing academic discourse, exemplified by works like Banik, Zarychta [16], which introduced dual conceptual frameworks explicating the intricate interplay between social support and self-efficacy. Zhang, Hasibagen [14] deepened this discourse by highlighting the intermediating influence of self-efficacy in the continuum of social support to PA behaviour. The prevailing theory suggests that the affirmation and motivation derived from robust social support networks serve to fortify individual confidence, thereby enhancing self-efficacy. Furthermore, the emotional bolstering and practical solutions provided through social support pave the way for reinforced individual resilience, adding further impetus to self-efficacy.

### 4.2 Self-efficacy’s impact on PA and health

Self-efficacy, as a psychological construct, plays a pivotal role in influencing individual PA behaviours, a phenomenon consistently underscored in empirical research. Our study further reinforces this narrative, underscoring the significant influence of self-efficacy on PA—a sentiment echoed across numerous preceding investigations. Indeed, both longitudinal and cross-sectional studies consistently demonstrate a positive connection between self-efficacy and various aspects of PA, such as frequency, duration, and intensity. Delving into our observations, it becomes apparent that heightened self-efficacy not only initiates participation but also fosters sustained commitment to physical regimens. This theme aligns with works by Cataldo, John [22] and Horcajo, Santos [23], both of which emphasise self-efficacy’s critical role in determining physical fitness outcomes. At a more detailed level, self-efficacy functions as the bedrock upon which individual goals and aspirations are built, motivating them to set and achieve ambitious physical milestones. Beyond goal-setting, self-efficacy equips individuals with an arsenal of resilience and problem-solving prowess, thereby instilling a proactive and solution-centric demeanour when confronted with physical challenges. These empirical findings vividly illustrate the inseparable connection between self-efficacy, PA and overall fitness. As we progress, understanding the nuances of this relationship holds profound implications for crafting interventions aimed at enhancing physical fitness.

### 4.3 PA’s influence on physical fitness

The intricate relationship between PA and physical fitness has long captivated scholars and health professionals, given its profound implications for health outcomes and disease prevention. Our investigation reaffirms the prevailing consensus: higher levels of PA are robust predictors of superior physical fitness. Numerous studies support this correlation [24–26]. The exhaustive review of Rauner, Mess [27] shed light on the nature of this relationship among adolescents, highlighting the enduring interest in this research area. Similarly, Gea-García, González-Gálvez [28] explored the interplay between PA and fitness among physical education students, advocating for the role of activity in preventing chronic diseases and fostering holistic health. Echoing these studies, our results suggest that the benefits associated with PA might arise from mechanisms such as enhanced metabolic rates—potentially driven by consistent muscle engagement—improved muscle and bone resilience, honed coordination, and cardiovascular health. Interestingly, our study unveiled a gender-based dimension, with female participants outshining males. This difference might be attributed to females’ preference for specific types of exercise, like aerobic workouts that optimise cardiovascular health and weight management. Additionally, females are more likely to engage in group fitness classes such as yoga and pilates, which not only improve flexibility and strength but also foster a sense of community and motivation. Influencing factors may also include dietary choices, with females possibly gravitating towards nutrient-dense diets and heightened health awareness, resulting in behaviours that enhance fitness test scores. Furthermore, females often exhibit higher levels of health consciousness, leading to more consistent participation in regular physical activity and adherence to fitness routines. Another possible reason is the influence of social support systems. Females might receive more encouragement from peers and family members to maintain a healthy lifestyle. This support can play a crucial role in sustaining long-term fitness habits. While our results are insightful, they underscore the necessity of broader samples to examine these gender-based nuances meticulously.

### 4.4 Mediation of PA and self-efficacy

Mediation, in research, refers to a process where a third variable elucidates the relationship between an independent and a dependent variable. In this study, we hypothesised that PA would serve as this mediating factor, explaining the relationship between social support and adolescent physical fitness. Our empirical data supports this assertion, demonstrating that social support directly influences physical activity levels, which in turn correlates with adolescent physical fitness. Through the lens of physical activity levels, social support indirectly contributes to enhanced adolescent physical fitness. This perspective aligns with previous works, including Ren, Hu [10] and Banik, Zarychta [16], which proposed the intertwined relationship between social support, activity, and physical fitness. Our findings, building on this existing knowledge base, provide a nuanced understanding, foregrounding the complex dynamics at play.

Similarly, we hypothesised that social support, by strengthening self-efficacy, would indirectly enhance physical fitness. The collected data resonate with this assertion, highlighting the symbiotic relationship between social support and self-efficacy, which subsequently impacts physical fitness. Self-efficacy, in this context, functions as the pivotal mediator between social support and physical fitness. Such findings echo prior research by Yiming, Shi [29] and provide empirical support for the significant role of self-efficacy in promoting adolescent physical fitness. Recognising self-efficacy’s significant impact—both directly and indirectly—on fostering PA, as corroborated by studies such as Banik, Zarychta [16] and Bateman, Myers [30], implies that to fully harness the potential of social support, adolescents should focus on enhancing their self-efficacy. These insights hold significant potential for developing interventions and policy guidelines aimed at bolstering adolescent physical fitness.

### 4.5 Strengths, limitations and future research directions

By elucidating the relationships between social support, PA, self-efficacy, and physical fitness, this study offers both empirical validation and novel theoretical insights. The findings contribute to the current understanding of how social support can enhance self-efficacy, which in turn promotes higher levels of physical activity and better physical fitness outcomes. This aligns with Bandura’s Social Cognitive Theory, which posits that self-efficacy is a critical determinant of behaviour change, and underscores the importance of social influences in shaping health behaviours. Moreover, the study extends existing literature by demonstrating the specific pathways through which social support influences physical activity and fitness, providing a more nuanced understanding of these dynamics. It also highlights the role of gender differences, suggesting that interventions may need to be tailored to address unique motivational and social factors affecting males and females differently. However, its reliance on self-reported data, a narrow demographic scope from a single Chinese university, and the use of convenience sampling underscore methodological constraints that may limit its generalizability and introduce biases. Future research trajectories, to enhance precision and representation, should pivot towards objective evaluative tools, such as physiological markers or third-party evaluations, span China’s diverse landscape, and embrace more refined sampling methods. Given the intriguing gender-based variations observed, a deeper exploration into such dynamics is warranted, as is a rigorous analysis of mediation structures potentially revealing further latent variables that intricately intertwine social support, activity, self-efficacy, and physical fitness together. Such analyses could involve structural equation modeling to identify and confirm the pathways and interactions between these variables, providing a more comprehensive theoretical framework. Moreover, future studies should explore the long-term effects of social support and self-efficacy on physical activity and fitness outcomes, and investigate the role of cultural, social, and environmental factors in shaping these relationships.

## 5. Conclusion

In a pioneering exploration based on the YPAP model, this study unveils the intricate mechanisms through which social support influences adolescents’ physical fitness within a Chinese cultural context. Notably, our findings confirm that social support stands as a robust predictor of both PA and self-efficacy. Moreover, self-efficacy emerges as a salient factor, substantially enhancing PA and, consequently, elevating physical fitness among adolescents. Furthermore, our results demonstrate that PA directly contributes to elevated physical fitness. Crucially, both self-efficacy and PA play mediating roles in the relationship between social support and fitness outcomes. As we chart the path forward, intervention strategies aiming at enhancing adolescent physical fitness should judiciously integrate initiatives to bolster social support, promote PA, and nurture self-efficacy.

## Supporting information

S1 Data Open access data file. (XLSX)
